# Supplementary material for: A deep learning method for HLA imputation and trans-ethnic MHC fine-mapping of type 1 diabetes
Source: Nat Commun. 2021 Mar 12;12:1639. doi: 10.1038/s41467-021-21975-x (PMC7955122; doi:10.1038/s41467-021-21975-x)
Supplement: Supplementary file 2 — Description of Additional Supplementary Files [file 41467_2021_21975_MOESM2_ESM.pdf]

Description of Additional Supplementary Files

File name: Supplementary Data 1.

Description: Detailed association results of the HLA variants in the MHC region on T1D.

File Name: Supplementary Data 2.

Description: Stepwise conditional association results in HLA-DRB1, -DQA1, and -DQB1 on T1D risk.

File Name: Supplementary Data 3.

Description: A correspondence table of amino acid polymorphisms and 4-digit classical HLA alleles is provided in an excel file format. It includes 4-digit classical alleles with a frequency >1% in either Europeans or Japanese populations based on the reference panels.
