# Supplementary material for: A deep learning method for HLA imputation and trans-ethnic MHC fine-mapping of type 1 diabetes
Source: Nat Commun. 2021 Mar 12;12:1639. doi: 10.1038/s41467-021-21975-x (PMC7955122; doi:10.1038/s41467-021-21975-x)
Supplement: Supplementary file 4 — Reporting Summary [file 41467_2021_21975_MOESM4_ESM.pdf]

nature research

Corresponding author(s): Yukinori Okada

Last updated by author(s): Jan 29, 2021

Reporting Summary

Nature Research wishes to improve the reproducibility of the work that we publish. This form provides structure for consistency and transparency in reporting. For further information on Nature Research policies, see our Editorial Policies and the Editorial Policy Checklist.

Statistics

For all statistical analyses, confirm that the following items are present in the figure legend, table legend, main text, or Methods section.

n/a	Confirmed	
☐	☒	The exact sample size (n) for each experimental group/condition, given as a discrete number and unit of measurement
☐	☒	A statement on whether measurements were taken from distinct samples or whether the same sample was measured repeatedly
☐	☒	The statistical test(s) used AND whether they are one- or two-sided<br>*Only common tests should be described solely by name; describe more complex techniques in the Methods section.*
☐	☒	A description of all covariates tested
☐	☒	A description of any assumptions or corrections, such as tests of normality and adjustment for multiple comparisons
☐	☒	A full description of the statistical parameters including central tendency (e.g. means) or other basic estimates (e.g. regression coefficient) AND variation (e.g. standard deviation) or associated estimates of uncertainty (e.g. confidence intervals)
☐	☒	For null hypothesis testing, the test statistic (e.g. F, t, r) with confidence intervals, effect sizes, degrees of freedom and P value noted<br>*Give P values as exact values whenever suitable.*
☐	☒	For Bayesian analysis, information on the choice of priors and Markov chain Monte Carlo settings
☒	☐	For hierarchical and complex designs, identification of the appropriate level for tests and full reporting of outcomes
☐	☒	Estimates of effect sizes (e.g. Cohen's d, Pearson's r), indicating how they were calculated

Our web collection on statistics for biologists contains articles on many of the points above.

Software and code

Policy information about availability of computer code

Data collection	No software was used.
Data analysis	We used publicly available software for the data analysis (Pytorch (1.4.1), SNP2HLA (v1.0.3, and Beagle (3.0.4) intrinsically), HIBAG (1.22.0.), Eagle (version 2.3), Minimac3 (version 2.0.1), and Chimera (version 1.14)). The source code of our method is available at https://github.com/tatsuhikonaito/DEEP-HLA. All our runtime analyses were performed on a dedicated server running CentOS 7.2.1511, with 48 CPU cores (Intel ® Xeon ® E5-2687W v4 @ 3.00 GHz) and 256 GB of RAM without GPU or a machine with Ubuntu 16.04.6 LTS with 20 CPU cores (Intel ® Core ™ i9-9900X @ 3.50 GHz), 2 GPUs (NVIDIA ® GeForce ® RTX 2080 Ti), and 128 GB of RAM.

For manuscripts utilizing custom algorithms or software that are central to the research but not yet described in published literature, software must be made available to editors and reviewers. We strongly encourage code deposition in a community repository (e.g. GitHub). See the Nature Research guidelines for submitting code & software for further information.

Data

Policy information about availability of data

All manuscripts must include a data availability statement. This statement should provide the following information, where applicable:

- Accession codes, unique identifiers, or web links for publicly available datasets
- A list of figures that have associated raw data
- A description of any restrictions on data availability

The Japanese HLA data have been deposited at the National Bioscience Database Center (NBDC) Human Database with the research ID hum0114 (https://humandbs.biosciencedbc.jp/en/hum0114-v2). Independent HLA genotype data of Japanese population have been deposited at the NBDC with the research ID hum0028 (https://humandbs.biosciencedbc.jp/hum0028-v2) and available through the Japanese Genotype-phenotype archive (JGA) with the accession ID JGAS000018 (https://ddbj.nig.ac.jp/resource/jga-study/JGAS000018). T1DGC HLA reference panel can be download at a NIDDK central repository with a request

(https://repository.niddk.nih.gov/studies/t1dgc-special/). GWAS data of the BBJ are available at the NBDC Human Database with the research ID hum0014 (https://humandbs.biosciencedbc.jp/hum0014-v18). The analysis of UKB GWAS data was conducted via the application number 47821 (https://www.ukbiobank.ac.uk/). The protein structures of HLA-A, HLA-DR, and HLA-DQ are available on Protein Data Bank entries 2BVP (https://www.rcsb.org/structure/2BVP), 3PDO (https://www.rcsb.org/structure/3PDO), and 1UVQ (https://www.rcsb.org/structure/1UVQ), respectively.

Field-specific reporting

Please select the one below that is the best fit for your research. If you are not sure, read the appropriate sections before making your selection.

- [x] Life sciences
- [ ] Behavioural & social sciences
- [ ] Ecological, evolutionary & environmental sciences

For a reference copy of the document with all sections, see nature.com/documents/nr-reporting-summary-flat.pdf

Life sciences study design

All studies must disclose on these points even when the disclosure is negative.

Sample size	For the model training and robust benchmarking, we used two different HLA imputation reference panels: (1) our Japanese reference panel of 1,120 individuals (2 were removed after QC), and (2) T1DGC reference panel of 5,225 individuals (103 were removed after QC). This sample size is considered to be sufficient to obtain reliable accuracy of HLA imputation. Independent Japanese data consisted of 908 individuals. For trans-ethnic MHC fine-mapping, 62,387 individuals from BBJ and 354,459 individuals from UKB were included.
Data exclusions	We excluded 2 (our Japanese panel) and 103 (T1DGC panel) individuals' data in which sides of some HLA alleles were discordant among different resolutions after pre-phasing, which are not consistent with diploid genome structure.
Replication	For benchmarking our imputation method, we used 10-fold cross-validation (randomly assigned) in constructing Japanese and European models respectively. The mean of accuracy metrics were used for their representative values. In addition, for Japanese model, we evaluated its performance when it was applied to an independent dataset.
Randomization	In 10-fold cross-validation, we randomly split individual data.
Blinding	The design of our study did not include any clinical interventions, which need consideration of blinding.

Reporting for specific materials, systems and methods

We require information from authors about some types of materials, experimental systems and methods used in many studies. Here, indicate whether each material, system or method listed is relevant to your study. If you are not sure if a list item applies to your research, read the appropriate section before selecting a response.

Materials & experimental systems

n/a	Involved in the study	
x		Antibodies
x		Eukaryotic cell lines
x		Palaeontology and archaeology
x		Animals and other organisms
	x	Human research participants
x		Clinical data
x		Dual use research of concern

Methods

n/a	Involved in the study	
x		ChIP-seq
x		Flow cytometry
x		MRI-based neuroimaging

Human research participants

Policy information about studies involving human research participants

Population characteristics	For 62,387 participants from BBJ, average age: 61.9 yr, 48.0% males and 52.0% females, 831 T1D cases and 61,556 controls. For 354,459 participants from BBJ, average age: 56.8 yr, 46.6% males and 53.4% females, 732 T1D cases and 353,727 controls. The detailed information of the participants is described in the previous literatures [BBJ: Hirata, M. et al. J. Epidemiol. 27, S9–S21 (2017), and Nagai, A. et al. J. Epidemiol. 27, S2–S8 (2017) and UKB: Sudlow, C. et al. PLoS Med. 12, 1–10 (2015), and Bycroft, C. et al. Nature 562, 203–209 (2018)].
Recruitment	The BBJ project is a population-based cohort study that recruited approximately 200,000 participants with the diagnosis of at least one of 47 diseases from 12 medical institutions in Japan. The UKB project is a population-based cohort study that recruited approximately 500,000 people aged between 40 and 69 yr from 2006 to 2010 from across the United Kingdom. There exist no self-selection biases that can potentially affect the results of the case-control association study (e.g. genotype data of individuals).
Ethics oversight	This study was approved by the ethical committee of Osaka University Graduate School of Medicine. All the participants provided written informed consent approved from ethics committees of RIKEN Center for Integrative Medical Sciences, and the Institute of Medical Sciences, the University of Tokyo.

Note that full information on the approval of the study protocol must also be provided in the manuscript.
